# Implementing the Smile4life intervention for people experiencing homelessness: a path analytical evaluation

**DOI:** 10.1186/s12903-021-01747-1

**Published:** 2021-08-05

**Authors:** Laura Beaton, Gerry Humphris, Andrea Rodriguez, Ruth Freeman

**Affiliations:** 1 Dental Health Services Research Unit, School of Dentistry, University of Dundee, Dundee, DD1 4HN UK; 2 School of Medicine, University of St Andrews, St Andrews, KY16 9TF UK

**Keywords:** Oral health, Homeless persons, Questionnaires

## Abstract

**Background:**

People experiencing homelessness have high levels of dental decay, oral cancer and poor oral health-related quality of life. The Scottish Government sought to address these issues by developing a national oral health improvement programme for people experiencing homelessness, named Smile4life. The aim was to investigate implementation behaviours and the role of work-related beliefs upon the delivery of the Smile4life programme across NHS Board areas in Scotland.

**Methods:**

Non-probability convenience sampling, supplemented by snowball sampling, was used to recruit practitioners working across the homelessness sector. The overall evaluation of the implementation of the Smile4life programme was theoretically informed by the Behaviour Change Wheel. The questionnaire was informed by the Theoretical Domains Framework and was divided into three sections, demography and Smile4life Awareness; Smile4life Activities; and Smile4life work-related beliefs. A psychometric assessment was used to develop Smile4life Awareness, Smile4life Activities, Ability to Deliver and Positive Beliefs and Outcomes subscales. The data were subjected to K-R20, exploratory factor analysis, Cronbach’s alpha, t-tests, ANOVA, Pearson’s correlation analysis and a multivariate path analysis.

**Results:**

One hundred participants completed the questionnaire. The majority were female (79%) and worked in NHS Boards across Scotland (55%). Implementation behaviour, constructed from the Delivering Smile4life scale and the summated Smile4life activities variable, was predicted using a linear model a latent variable. The independent variables were two raw variables Positive Beliefs and Outcomes, and Ability to deliver Smile4life. Results showed relatively good model fit (chi-square (1.96; *p* > 0.15), SRMR (< 0.08) and R^2^ (0.62) values). Positive and highly significant loadings were found describing the Implementation Behaviour latent variable (0.87 and 0.56). The two independent variables were associated (*p* < 0.05) with Implementation Behaviour.

**Conclusions:**

Work-related factors, such as positive beliefs and outcomes and ability to deliver are required for implementation behaviours associated with the delivery of the Smile4life programme. Future work should include training centred on the specific needs of those involved in the homelessness sector and the development of accessible training resources, thereby promoting implementation behaviours to assist the progression and sustainability of the Smile4life programme.

**Supplementary Information:**

The online version contains supplementary material available at 10.1186/s12903-021-01747-1.

## Background

In Scotland in 2019/20, there were 36,855 homelessness applications [1]. Of these 30,146 (96%) were considered as unintentionally homeless, defined as an individual or household has become homeless due to factors beyond their control, e.g. illness, or financial difficulties such as redundancy [2]. From these applications, 31,333 households were assessed as homeless and 11,665 people were residing in temporary accommodation. This represented a rise of 4% of homeless households and an increase of 6% of those in temporary accommodation compared with 2018–2019 [1].

Moreover, people experiencing homelessness may also be facing social exclusion. Social exclusion is a “dynamic, multidimensional and relational process” which can involve interpersonal, societal and political factors, such as poverty, illness, and geographical isolation [3, 4]. For those experiencing social exclusion in addition to homelessness, their oral health was typified by a greater prevalence of dental decay experience, oral cancer and poorer oral health-related quality of life [3–5]. This may be explained by a number of factors: people experiencing homelessness have been found to be irregular or emergency-only dental attenders [6, 7], and may have poorer oral hygiene and more negative health behaviours when compared to the general population, e.g. increased smoking, alcohol consumption and substance misuse [7, 8]. In addition, the limited utilisation of dental services by people experiencing homelessness may be due, in some instances, to the precarious living conditions associated with homelessness, characteristics of the healthcare system (e.g. inflexibility regarding appointments) and socio-political phenomena [3, 7].

Acknowledging this ‘cliff-edge’ in oral health-health inequity, the Scottish Government published two seminal policy documents: The Dental Action Plan, and the Health and Homelessness Standards [9, 10]. By 2007, as part of the Dental Action Plan, the Scottish Government conceived and developed its national oral health improvement programme for people experiencing homelessness, named Smile4life. The Smile4life Guide for Trainers and the behaviour change intervention [11], the cornerstone of the programme, was underpinned by a normative need survey [5, 12] together with a qualitative exploration of expressed and felt needs of people experiencing homelessness in Scotland [12, 13].

Following the 2012 launch of the Smile4life Guide for Trainers and intervention, a process evaluation was conducted, to evaluate how Smile4life was being adopted and implemented in Scottish NHS Boards [14]. Underpinned by Rogers’ Theory of Diffusion of Innovations [15], this process evaluation demonstrated variations in practice across the NHS Boards. Some NHS Boards quickly adopting the intervention, while others struggled, affected by numerous barriers, including a lack of resources [14]. While this original process evaluation highlighted factors affecting the Boards’ adoption and delivery of the Smile4life programme, what remained unanswered were the role of Smile4life practitioners’ beliefs and routine practices regarding the implementation of Smile4life. More recent work supported the need to examine work-related beliefs and behaviours, suggesting that the interplay between organisational factors with skills and beliefs could affect working behaviours of those implementing Smile4life [14, 16].

Therefore, the aim of this survey was to investigate implementation behaviours and the role of work-related beliefs upon the delivery of the Smile4life programme across NHS Board areas in Scotland, using a path analytical approach.

## Methods

### Study setting

This survey took place in Scotland and the participants were those working within the homelessness sector or within NHS Boards where they were associated with services for people experiencing homelessness.

### Sample and recruitment

Non-probability convenience sampling was used for the initial stage of recruitment [17]. Twenty-three NHS Smile4life practitioners were contacted by email. Snowball sampling was then used to supplement. This overall sampling procedure was chosen to recruit hard-to-reach practitioners and to invite them to participate [18]. Potential participants included NHS practitioners, Third Sector, Local Authority staff, people who had received Smile4life training and those involved in Smile4life in some capacity.

### Theoretical base

This investigation was theoretically informed by the Behaviour Change Wheel (BCW) [19, 20]. The BCW was chosen as it provides a guide and a way of evaluating intervention implementation. In addition, it is a synthesis of all current behavioural change frameworks and as such is applicable to ‘any behaviour in any setting’ [20]. Moreover, since the BCW connects to the Theoretical Domains Framework (TDF), it provides a more detailed understanding of behaviour, and can be used to examine determinants of behaviour [21, 22]. Therefore, the BCW was adopted using its evaluation process to inform this investigation because it is inclusive of the majority of factors affecting behaviour change.

### The questionnaire

For the purposes of questionnaire development, a panel of five experts (representing dental public health, health psychology, homelessness research and policy, special care dentistry, and behaviour change research) was convened to provide feedback on the questionnaire, particularly the use of the 14 TDF domains [23–26]. The panel provided feedback on the question wording and agreed with the authors on the removal of four TDF domains from the questionnaire. This resulted in employing nine domains with two items each, one domain with three items, and therefore 21 items in total for the work-related belief section (Section 3) of the questionnaire.

The questionnaire was divided into three sections.

- Section 1: Demography and Smile4life Awareness. The first section enquired of the respondents’ age, gender, sector and job title, together with their awareness of the Smile4life Guide for Trainers and Intervention. Four questions assessed Smile4life awareness, e.g. ‘Have you read the Guide for Trainers?’ and were in a yes (scoring 1)/no (scoring 0) format.
- Section 2: Smile4life Activities. Smile4life Activities were assessed using a yes (scoring 1)/no (scoring 0) format. Five work-related Smile4life items e.g. ‘delivering Smile4life training’, were presented to the respondents to indicate which of Smile4life activities they had participated.
- Section 3: Smile4life work-related beliefs. This section consisted of the 10 domains within the TDF and consisted of the 21 work-related belief items, such as beliefs within the domains of knowledge, skills, capabilities, intentions, etc. The work-related belief items were scored on a 5-point Likert scale with scores ranging from 5 (strongly agree) to 1 (strongly disagree).

All scores from the 4 items of the Smile4life Awareness checklist were subjected to a Kuder-Richardson 20 (KR20) test to determine the reliability of the items to form a consistent scale. The KR20 score for all the Smile4Life Awareness items was α = 0.77, indicating that the individual items had satisfactory internal consistency to form a reliable scale [27]. The new Smile4life Awareness Scale scores ranged from 0 (no awareness) to 4 (awareness), with mean scores of 2.41 (± 1.40). Similarly, all scores from the 5 work-related items from Smile4life Activities were subjected to the KR20 to assess reliability to form a consistent scale. The KR20 score for the 5 items was α = 0.75, indicating the items were satisfactorily internally consistent and formed a reliable scale [27]. The Smile4life Activities Scale scores ranged from 0 (none) to 5 (all behaviours) with mean scores of 1.62 (± 1.60).

All scores from 20, (excluding the item, ‘Delivering Smile4life is part of my regular practice when engaging with service users’), of the 21 work-related beliefs were subjected to principal component analysis with varimax rotation in order to cluster items to form consistent and reliable scales. Table 1 presents the individual items and scales together with their Cronbach’s alpha as a measure of scale reliability (internal consistency). Three scales were derived, which explained 62.5% of the variance. Subscales 1 (α = 0.94) and 2 (α = 0.87) had satisfactorily internal consistency and formed reliable scales. The Cronbach alpha score, however, for the third subscale indicated that this subscale had poorer internal consistency (α = -0.03). This indicated that it could not form a reliable scale. It was subsequently excluded from further analysis. Subscale 1 was named ‘Ability to deliver Smile4life’ and Subscale 2 was named ‘Positive beliefs and outcomes’.

### Data collection

The questionnaire was hosted online using Jisc Online Surveys (https://www.onlinesurveys.ac.uk/). Two reminders were sent to the original list of contacts and the questionnaire was advertised on the Scottish Dental website (https://www.scottishdental.org/) and in the Faculty of Homeless and Inclusion Health’s Scottish newsletter. In addition to the online version of the questionnaire, hard copies were distributed at appropriate meetings and events where Smile4life practitioners, or indeed any practitioner working on oral health and homelessness, were present.

### Data analysis

Quantitative data from completed questionnaires were compiled into a single data work file with the data exported from Jisc Online Surveys into IBM SPSS Statistics v24 and STATA v16. Age was split into three age categories: younger (16–30 years = 0), middle (31–50 years = 1) and older (51 + years = 2). Gender was coded as a dichotomous variable (female = 0: male = 1). Working sector was divided into 4 categories NHS (= 0); Local authority (= 1); Third Sector/NGO/Charity (= 2), other (= 3). The data were subjected to descriptive analysis, K-R20, exploratory factor analysis, Cronbach’s alpha, t-tests, ANOVA, Pearson’s correlation analysis and a multivariate path analysis. The path model approach included a latent variable named ‘Implementation Behaviour’ defined from the two behavioural raw variables: ‘Delivery of Smile4life is part of my regular practice’ and the sum of the Smile4life activities. A small set of independent variables were chosen to enter the path model determined by the relatively small sample size. The chi-square (*p* > 0.1) and the standardized root mean squared residual (SRMR < 0.08) was inspected to determine model fit appropriate for linear models including raw and latent variables. The alpha level was 0.05. Two-sided tests were employed throughout.

## Results

One hundred practitioners completed the questionnaire, over a six-month data collection period between March – August 2019. The majority were female (79%). The age range of participants was from 18 to 65 years with 74% aged between 31 and 60 years of age. Over half of participants (55%) reported that they worked within NHS Boards, with 32% in the Third Sector or NGOs, 13% in other localities e.g. community pharmacies and 5% in local authorities. Areas of work included, dental services (44%), community support and development (29%), community health (11%) with the remaining working in other areas or in education.

### Smile4life awareness

Overall 33% of the participants were fully aware of the Smile4life programme, whereas 10% reported they had no awareness of it. 89% of the total sample reported that they knew of the Smile4life Guide for Trainers, with over half stating they had read the Smile4life Report (57%) and the Guide for Trainers (51%). 43% stated that they had received Smile4life training. 22% reported that they had known of the Smile4life programme in 2012, when the Guide for Trainers was launched. 40% of participants were unaware of when the Smile4life programme was implemented in their NHS Board area.

The total mean score for Smile4life Awareness was, 2.41(SD 1.41). Smile4life Awareness was significantly explained by the grouping variables, age (F[2,96] = 7.51: *p* < 0.001) and working sector (F[3,96] = 22.00: *p* < 0.001). Therefore, participants in the 16–30 years age group had significantly lower total mean scores for Smile4life Awareness than those in the 31–50 or 51 + age groups (Table 2). Participants working in NHS Boards (3.22, SD 1.13) had significantly greater mean scores than those working in local authorities (1.6, SD 1.52) NGOs (1.23, SD 0.82) or Other Sectors for Smile4life Awareness. There were no significant differences between female (2.51, SD 1.42) and male (2.05, SD 1.36) total mean scores for Smile4life Awareness (t = 1.34: *p* = 0.18).

### Smile4life activities

Overall, only 3% of participants participated in all five Smile4life actions. The list of these activities can be found in Additional File 1. Thirty-eight stated they did not participate in any Smile4life activity. The individual Smile4life Activities most commonly stated were, signposting people experiencing homelessness for oral health care (55%), distributing toothbrush and toothpaste packs (43%) and other Smile4life-related activities, such as a management role in organising oral health promotion activities at NHS Board levels (35%).

The total mean score for Smile4life Activities was 1.62 (SD 1.60). Smile4life Activities was significantly explained by the grouping variable age. Those participants in the 16–30 years age group had significantly lower total mean scores for Smile4life Activities than those in the 31–50 or 51 + age groups (Table 2). Female participants (1.79, SD 1.62) had significantly larger total mean scores for Smile4life Activities than male participants (0.95, SD 1.35) (t = 2.07: *p* = 0.04). The grouping variable working sector did not explain differences in total mean Smile4life Activities scores (F[3,96] = 2.02 = *p* = 0.12).

**Table 1:** Smile4life work-related beliefs: subscales (means, SD, and reliabilities) and items (means, SD and factor loadings)

TDF domains	Items	Cronbach alpha	Factor loadings	Mean (SD)
	**Subscale 1: Ability to deliver Smile4life3**	0.94		26.46 (7.99)
Knowledge	I know how to deliver Smile4life.		0.90	3.18 (1.32)
Skills	I have the skills required to deliver Smile4life.		0.89	3.33 (1.36)
Skills	I have received sufficient training to help me deliver Smile4life.		0.90	3.03 (1.39)
Capabilities	I am confident that I can deliver Smile4life.		0.88	3.25 (1.35)
Capabilities	I find it easy to deliver Smile4life.		0.76	2.95 (1.20)
Intentions	I intend to put every effort I can into delivering Smile4life as outlined in the Guide for Trainers.		0.58	3.64 (1.08)
Social influences	I feel appreciated when I deliver Smile4life to service users.		0.50	3.41 (0.77)
Emotion	I find it rewarding to deliver Smile4life.		0.60	3.66 (0.90)
	**Subscale 2: Positive beliefs and outcomes**	0.88		28.79 (4.75)
Knowledge	I am familiar with oral health problems in people experiencing homelessness.		0.64	4.16 (0.99)
Consequences	I believe that Smile4life makes a difference to people experiencing homelessness.		0.84	4.04 (1.01)
Consequences	I believe what Smile4life aims to do is achievable.		0.61	2.23 (1.08)
Intentions	I intend to continue incorporating Smile4life into my work with service users.		0.63	3.77 (1.00)
Goals	Smile4life is a priority for me personally.		0.46	3.55 (0.99)
Reinforcement	It benefits me to implement Smile4life		0.46	3.48 (0.98)
Social Influences	I feel supported by my employer when delivering Smile4life.		0.52	3.42 (0.77)
Emotion	I feel pleased when I hear Smile4life success stories from homeless service users.		0.74	4.15 (0.91)

### Smile4life work-related belief scales

The mean total scores for the Ability to Deliver Smile4life scale were 26.46 (SD 8.04). The grouping variable age significantly explained differences in total mean scores for Ability to Deliver Smile4life. Those in the youngest age group had significantly lower total mean scores for the Ability to Deliver Smile4life compared with those in the middle or older age groups (Table 2). The grouping variable working sector significantly explained differences in total mean scores for Ability to Deliver Smile4life (F[3,96] = 10.63: *p* < 0.001). Therefore, those working in the NHS Boards (30.07, SD 7.13) had significantly greater mean scores than those working in local authorities (22.00, SD 7.18), NGOs (22.11, SD 7.14) or Other Sectors (21.92, SD 6.66). There were no significant differences between female (26.83, SD 8.27) and male (25.04, SD 7.14) total scores for Ability to Deliver Smile4life (t = 0.90: *p* = 0.37).

The mean total scores for Positive Beliefs and Outcomes were 28.79 (SD 4.75). The grouping variable age significantly explained differences in total mean scores for Positive Beliefs and Outcomes. Those in the youngest age group had significantly lower total mean scores for Positive Beliefs and Outcomes compared with those in the middle or older age groups (Table 2). The grouping variable working sector significantly explained differences in total mean scores for Positive Beliefs and Outcomes (F[3,96] = 3.22: *p* = 0.03). Multiple comparison tests, however, did not demonstrate any pair of comparisons to be significantly different. Likewise, there were no significant differences between female (28.92, SD 4.63) and male (28.28, SD 5.29) total scores for Positive Beliefs and Outcomes (t = 0.54: *p* = 0.58).

The total mean scores for the work-related belief scale, ‘Delivering Smile4life is part of my regular practice when engaging with service users’, were 3.00 (SD 1.12). The grouping variable age significantly explained differences in total mean scores for the Delivering Smile4life scale. Those in the youngest age group had significantly lower total mean scores for Delivering Smile4life compared with those in the middle or older age groups (Table 2). The grouping variable working sector did not significantly explained differences in total mean scores for Delivering Smile4life (F[3,96] = 0.71: *p* = 0.55). Similarly, there were no significant differences between female (3.05, SD 1.14) and male (2.81, SD 1.03) total mean scores for Delivering Smile4life (t = 0.88: *p* = 0.38).

Prior to multivariate analysis a Pearson’s correlation of all independent and dependent variables were examined (Table 3). There were numerous significant associations. Of note was the positive set of associations between age and the majority of the Smile4life variables. Gender shared little variance across the variable pool.

**Fig. 1:**
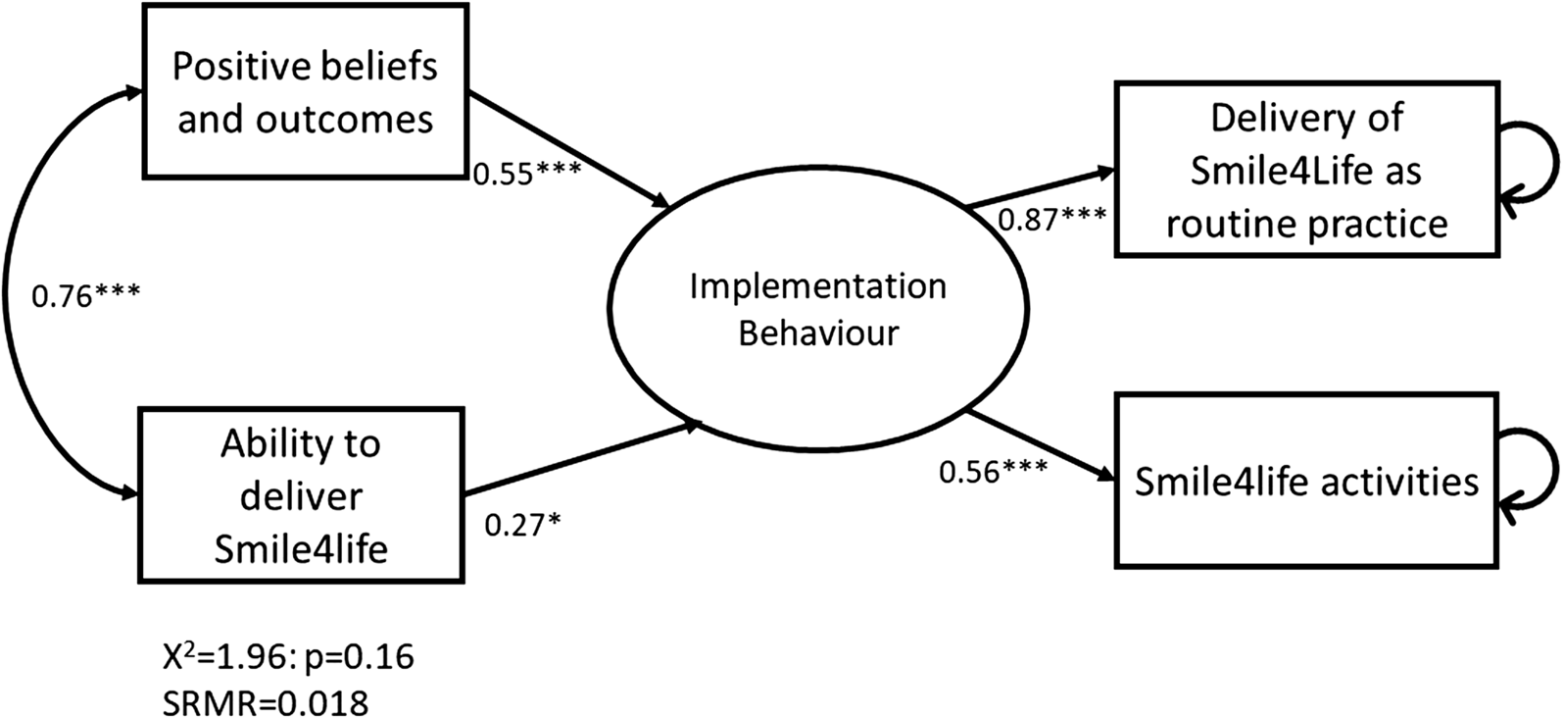
Path model of latent variable: Implementation Behaviour predicted by ‘positive beliefs and outcomes’ and ‘Ability to deliver Smile4life’ predicted by two independent raw variables. (Standardized parameter coefficients presented. Residual errors of raw behavioural variables indicated by single-arrowhead curves.)

To predict implementation behaviour using a linear model a latent variable was constructed from the Delivering Smile4life scale and the summated Smile4life activities variable. This was considered parsimonious with the advantage of testing a single model rather than running two separate regression models for each of the raw behavioural variables, which would increase the risk of Type II errors. Selection of independent variables was limited to two raw variables, namely Positive Beliefs and Outcomes, and Ability to deliver Smile4life. Age was not entered into the model as the univariate comparisons above had shown significant associations with all the substantive variables (independent and dependent) violating model independence assumptions. Gender of participant was not closely associated with any model variables and hence omitted. Results of the model showed relatively good model fit on inspection of the chi-square (1.96; *p* > 0.15), SRMR (< 0.08) and R^2^ (0.62) values. In addition, positive and highly significant loadings were found describing the Implementation Behaviour latent variable (0.87 and 0.56) (Fig. 1). The two independent variables were associated (*p* < 0.05) with Implementation Behaviour. Positive Beliefs and Outcomes was associated strongly with Implementation Behaviour (0.55). The residuals were small and non-substantive as indicated by the standardized root mean squared residual.

**Table 2:** Comparison of Smile4life Scales by age group of participants

Smile4life subscales	Younger age group (16–30 years) (x: SD)	Middle age group (31–50 years) (x: SD)	Older age group (51 + years) (x: SD)	F(df)	*p*
Smile4life Awareness	1.14^1^¥ (1.09)	2.58^2^ (1.32)	2.67^2^ (1.42)	7.51 (2,96)	< 0.001
Smile4life Activities	0.14^1^ (0.36)	2.06^2^ (1.59)	1.64^2^(1.58)	9.02 (2,96)	< 0.001
Ability to Deliver Smile4life	18.13^1^ (5.52)	27.00^2^ (8.34)	28.55^2^ (6.89)	8.71 (2,97)	< 0.001
Positive Beliefs and Outcomes	25.07^1^ (5.38)	29.21^2^ (4.79)	29.63^2^ (3.85)	5.53 (2,97)	0.005
Delivering Smile4life is part of my regular practice	2.21^1^ (0.89)	3.29^2^ (1.11)	2.29^2^ (1.07)	5.66 (2,97)	0.005

¥ Suffixes show the significant differences in the mean scores between groups with non-identical numeric characters

**Table 3:** Pearson’s Correlation matrix

	S4L awareness	S4L activities	Ability to deliver S4L	Positive belief and outcomes	Deliver S4L regular practice	Age	Gender
S4L awareness	1.00						
S4L activities	0.37*	1.00					
Ability to deliver S4L	0.72*	0.44*	1.00				
Positive belief and outcomes	0.53*	0.40*	0.75*	1.00			
Deliver S4L regular practice	0.31*	0.48*	0.56*	0.67*	1.00		
Age	0.29*	0.19*	0.32*	0.27*	0.12	1.00	
Gender	− 0.14	− 0.21*	− 0.09	− 0.06	− 0.09	0.01	1.00

*p < 0.05

## Discussion

This study aimed to investigate implementation behaviours and the role of work-related beliefs upon the delivery of the Smile4life programme across NHS Board areas in Scotland. It revealed that for the delivery of the Smile4life programme a wide range of practitioners from NHS Boards, Local Authority, and Third and other (e.g. Education) sectors was essential [14, 28]. A recent four-year research study pointed to the importance of multi-disciplinary working with homeless people across primary and mental health services to promote preventive dental care [29]. Moreover, Zucchero et al. [30] stressed the need for collaborations between sectors to enable effective programme implementation. These recent studies, together with a mixed method review on community-based interventions [31], support the findings here and confirm the requirement for a wide range of practitioners from various sectors to be involved in programmes such as Smile4life.

The final linear model explained 62% of the variation in Smile4life Implementation Behaviour. The advantage of devising the latent variable named ‘Implementation Behaviour’ was that it combined in one component, a broad range of Smile4life activities and the second component was concerned with Smile4life as simply the participants’ report of their *regular practice*.

Of particular importance was the equivalence of the strength of Positive Beliefs and Outcomes across practitioners working in NHS Boards and other sectors and its role as a predictor of Implementation Behaviour. Previous work conducted with Smile4life practitioners, regarding work-related attitudes suggested that those with more positive work-related beliefs were more successful when implementing Smile4life [14]. As Beaton et al. revealed, ‘the success of engagement. . was closely associated with the individual oral health practitioner, and their personality, experience, communication skills and how they perceived their job role and responsibilities’ [28]. Therefore, it was interesting to note that the role of the ability to deliver the Smile4life programme was not only predictive of Implementation Behaviours but was composed of work-related beliefs associated with appropriate knowledge, skills and training together with increased confidence to deliver the programme. It may be proposed that the construct, ‘ability to deliver Smile4life’, points to the need for tailored and appropriate training to promote the work-related skills and confidence [16] for those working in sectors other than the NHS Boards. While training may be supported by existing resources, other platforms and involving those with lived experience should be considered [3]. Doing so, it may be postulated, would sustain and progress the implementation of the Smile4life programme. Support for this proposition may be gleaned from the BCW that suggests that interventions should include training to develop practitioners’ opportunities and capabilities for implementation [20].

There were a number of limitations affecting this work, including the use of self-report measures for all variables. Recruitment of participants for this study was challenging, which encouraged the adoption of a snowball sampling technique in order to recruit hard-to-reach people working in the homelessness sector. Using this sampling approach and the relatively small number of respondents, caution is required in the interpretation of this analysis and its generalisability to other groups working in this field. Nevertheless, the findings do suggest that work-related factors, such as positive beliefs and outcomes and ability to deliver are required for implementation behaviours associated delivery of this programme.

## Conclusions

This study has revealed current practice and practitioners’ beliefs regarding the Smile4life intervention. By doing so, it was possible to extract recommendations that may be used to promote the future delivery of Smile4life, and support practitioners in their reported Smile4life-related work. Future steps should include training centred on the specific needs of those involved in the homelessness sector and the development of accessible training resources, thereby promoting implementation behaviours to assist the progression and sustainability of the Smile4life programme.

## Supplementary Information

**Additional file 1**. Overall, only 3% of participants participated in all five Smile4life actions.

## Data Availability

The dataset used and analysed during the current study is not available due to restrictions from the ethical committee. Please contact the corresponding author for more information.

## Abbreviations

NHS: National Health Service
BCW: Behaviour change wheel
TDF: Theoretical domains framework
KR20: Kuder–Richardson 20
ANOVA: Analysis of variance
SRMR: Standardized root mean squared residual
NGO: Non-Governmental Organisation
SD: Standard deviation
S4L: Smile4life
