# Supplementary material for: Implementing the Smile4life intervention for people experiencing homelessness: a path analytical evaluation
Source: BMC Oral Health. 2021 Aug 5;21:383. doi: 10.1186/s12903-021-01747-1 (PMC8340521; doi:10.1186/s12903-021-01747-1)
Supplement: Supplementary file 1 — Additional file 1. Overall, only 3% of participants participated in all five Smile4life actions. [file 12903_2021_1747_MOESM1_ESM.docx]

**Implementing the Smile4life intervention for people experiencing homelessness: a path analytical evaluation**

1. Laura Beaton, Dental Health Services Research Unit, School of Dentistry, University of Dundee, Dundee, United Kingdom, DD1 4HN (Corresponding Author)
2. Gerry Humphris, School of Medicine, University of St Andrews, St Andrews, United Kingdom, KY16 9TF
3. Andrea Rodriguez, Dental Health Services Research Unit, School of Dentistry, University of Dundee, Dundee, United Kingdom, DD1 4HN
4. Ruth Freeman, Dental Health Services Research Unit, School of Dentistry, University of Dundee, Dundee, United Kingdom, DD1 4HN

**Table S1 List of Smile4life activities**

| 1 | Train staff (NHS/NGO) in how to deliver oral health advice to people experiencing homelessness |
| --- | --- |
| 2 | Deliver one-to-one oral health advice to people experiencing homelessness |
| 3 | Signpost people experiencing homelessness to where they can get oral health care |
| 4 | Distribute toothbrush and toothpaste packs |
| 5 | Accompany people experiencing homelessness to the dentist |
